# The role of neoadjuvant chemotherapy for resectable colorectal liver metastases: a systematic review and meta-analysis

**DOI:** 10.18632/oncotarget.8671

**Published:** 2016-04-09

**Authors:** Wei Liu, Jian-Guo Zhou, Yi Sun, Lei Zhang, Bao-Cai Xing

**Affiliations:** ^1^ Hepatopancreatobiliary Surgery Department I, Key Laboratory of Carcinogenesis and Translational Research, Ministry of Education, Peking University School of Oncology, Beijing Cancer Hospital and Institute, Beijing, PR China; ^2^ Department of Oncology, Affiliated Hospital of Zunyi Medical University, Zunyi Medical University, Zunyi, China

**Keywords:** neoadjuvant, chemotherapy, colorectal liver metastases, meta-analysis

## Abstract

Neoadjuvant chemotherapy is being increasingly accepted as an effective treatment of resectable colorectal liver metastases (CRLM), but it may also damage the hepatic parenchyma. We performed a meta-analysis to compare the outcomes of patients who received neoadjuvant chemotherapy (NEO) prior to hepatic resection with hepatic resection without neoadjuvant chemotherapy (SG). Eligible trials were identified from Embase, PubMed, the Web of Science and the Cochrane library. Hazard ratios (HRs) with a 95% confidence intervals (CIs) were used to measure the pooled effect using a random-effects model. Statistical heterogeneity was detected by *I*^2^ test. Sensitivity analyses and publication bias were also assessed. The study outcomes included 3-year, 5-year disease-free and overall survival rate, respectively. Eighteen studies involving 6,254 patients were included. The pooled HRs for 5-year DFS and 5-year OS for NEO in the included studies calculated using the random-effects model were 1.38 (95 % CI; 1.26-1.51, *p*=0.00; *I*^2^=9.6%, *p*=0.36) and 1.19 (95% CI: 1.02-1.38; *p*=0.03; *I*^2^=49.2%, *p*=0.03), respectively. For CRLM patients with factors indicating a high risk of recurrence, the pooled HR for 5-year OS of NEO in the included studies calculated using the random-effects model was 0.69 (95% CI: 0.55-0.87; *p*=0.00; *I*^2^=0.0%, *p*=0.48). These results suggest neoadjuvant chemotherapy improved survival of patients with initially resectable CRLM and a high risk of disease recurrence.

## INTRODUCTION

Colorectal cancer (CRC) is the third most common cause of cancer-related deaths [1]. Metastasis is the major reason of mortality in CRC patients, with the liver being the only site of metastases in approximately 30% of the patients. Hepatic resection remains a well-accepted treatment modality for patients with colorectal liver metastases (CRLM) and is associated with 5-year survival rate ranging from 37% to 58% [2, 3].

Neoadjuvant chemotherapy has become an integral part of the multidisciplinary management of CRLM. Moreover, there is currently an increasing practice of administering neoadjuvant chemotherapy to patients with resectable CRLM, as it might increase the resectability of the hepatic lesions and treat occult metastases. Consistent with that idea, a recent review of 23 studies involving over 3,000 patients showed a benefit from neoadjuvant chemotherapy [4]. It has also been proposed neoadjuvant chemotherapy could be an effective component of individualized precision medicine for CRLM patients at high risk of disease recurrence. However, whether neoadjuvant chemotherapy is appropriate for patients with resectable CRLM remains controversial. Therefore, the aim of this meta-analysis was to assess the benefit of neo-adjuvant chemotherapy in the treatment of patients with primarily resectable CRLM.

## MATERIALS AND METHODS

### Search strategy

A comprehensive search was performed to identify all published studies on neoadjuvant chemotherapy administered to patients with resectable CRLM patients. Searches of the Embase, PubMed, Web of Science and Cochrane databases were conducted to identify eligible studies, with no language restriction. The keywords used for the search strategy were ‘colorectal liver metastases’ or ‘colonic liver metastases’ or ‘rectal liver metastases’ or ‘rectum neoplasm’ or ‘colon neoplasm’ and ‘liver resection’ or ‘hepatic resection’ and ‘neoadjuvuant chemotherapy’ or ‘preoperative chemotherapy’

### Inclusion and exclusion criteria

Included studies fulfilled the following criteria: (1) the study population were adults diagnosed with resectable CRLM; (2) the intervention was neoadjuvant chemotherapy administered prior to hepatic resection; (3) results were compared with patients undergoing hepatic resection without neoadjuvant chemotherapy; (4) outcomes included characteristics, overall survival (OS), disease-free survival (DFS), treatment-related complications and R1 resection rate.

The articles excluded from the analysis included (1) comments, editorials, systematic reviews and studies unrelated to our topics were excluded from the final analysis; (2) those that included patients with initially unresectable metastases; and (3) those in which the outcomes were not reported or were impossible to calculate for both groups. The quality of the studies was assessed independently by two investigators.

### Data extraction

Two independent investigators (Wei Liu and Jian-Guo Zhou) performed the abstract review and subsequent full text review. Disagreements between these two investigators were resolved through discussion until consensus was reached. A standardized data extraction form was used for the data extraction. The data extracted from the included studies were lead author; number of patients receiving neoadjuvant chemotherapy prior to hepatic resection (NEO) and the number receiving hepatic resection without neoadjuvant chemotherapy (SG); baseline patient characteristics, including tumor size>5cm, multiple metastases, site of primary cancer, primary lymph node status, synchronous CRLM and major hepatic resection; study region; recruitment period; 3-year and 5-year OS and DFS; R1 resection and treatment-related complication rate. Post-operative chemotherapy protocols were always based on the individual preferences of each institution.

### Quality assessment

A modified Newcastle-Ottawa scale (NOS) was used to assess the quality of the nonrandomized studies included in this meta-analysis [5]. This scale ranged from 0 to 9 points and consisted of three items that described the patient selection method, the comparability of the characteristics and the post-operative outcomes of the patients undergoing liver surgery for CRLM with or without neoadjuvant chemotherapy. Articles scored as ≥6 were deemed to be high-quality studies. The overall quality of the evidence and strength of recommendations were evaluated using GRADE [6]. GRADE Working Group evidence grades of evidence were as follows: high quality, further research is very unlikely to change our confidence in the estimate of effect; moderate quality, further research is likely to have an important impact on our confidence in the estimate of effect and may change the estimate; low quality, further research is very likely to have an important impact on our confidence in the estimate of effect and is likely to change the estimate; very low quality, we are very uncertain about the estimate.

### Statistical analysis

We assessed the overall efficacy of hepatic resection for CRLM patients based on the data from the included studies. For the time-to-event variables, the hazard ratios (HRs) for OS with 95%CIs were directly extracted or calculated using a calculation sheet as previously described [7]. The incidence of treatment-related death was treated as a dichotomous variable, and the number of deaths and the total number of patients were extracted from the included studies. Thereafter, the odds ratios (ORs) with 95% CI were calculated. Pooled estimates of the HRs and ORs were calculated using a random-effects model, regardless of heterogeneity. A test for heterogeneity, defined as the variation between individual trials for a given treatment, rather than that expected from chance, was used to assess whether the magnitude of a given treatment effect varied between the trials. The *I*^2^ statistic was used to describe the percentage of the total variation across studies caused by heterogeneity rather than chance. Heterogeneity was sonsidered substantial if a *I*^2^≥50% [8]. Meta-regression was conducted to determine the possible cause of region heterogeneity. The presence of publication bias was evaluated using Begg's and Egger's tests. Power calculation was performed after the studies had been collected using the methodology described by Cafri et al. [9]. Details on the macro and SAS code used are included in the online supplement material [10]. Values of *p* < 0.05 was considered to be significant. All statistical analyses were performed using STATA version 12.0 software (Stata Corporation, College Station, TX, USA).

## RESULTS

### Identification of eligible studies

**Figure 1:**
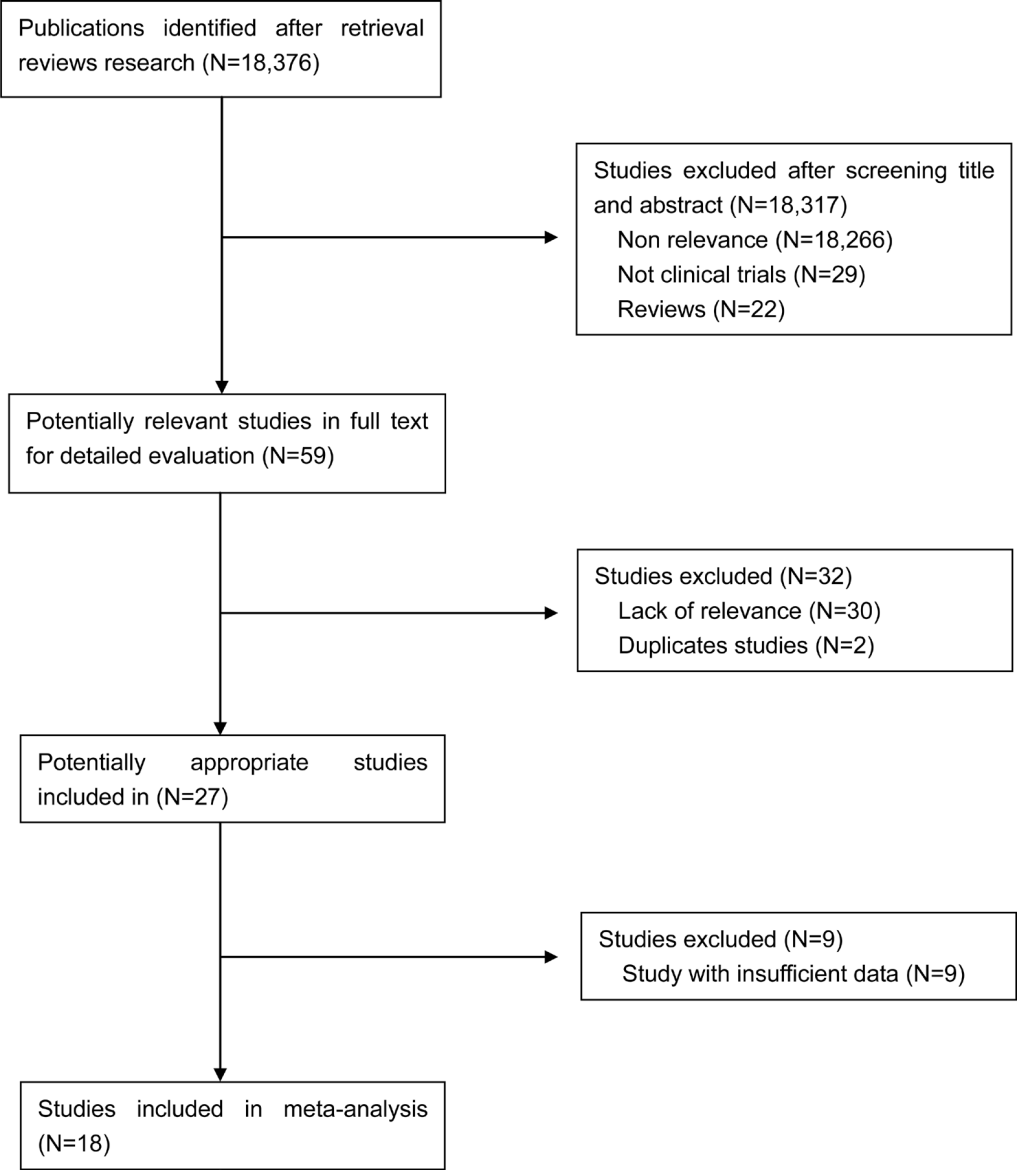
Flow chart for studies selection

A total of 18,376 CRLM-related citations were identified based on the initial search. After independent review, 18,358 studies were excluded ether because they were not relevant to the current analysis or they were ineligibility based on the inclusion criteria (Figure 1). Ultimately, eighteen studies (*n* = 6,254 patients) were included in this meta-analysis [11–28], among which four studies defined high risk factors for recurrence.

### Study characteristics

The included studies were published between 2003 and 2015. Among these studies, ten studies were conducted in Europe, four in America and four in Asia: two were conducted in Italy, two in the UK, one in Germany, one in Sweden, four in the USA, one in Japan, one in China, one in Korea, one in Israel and four in multiple centers of Europe. Fourteen comparisons focused on OS, and four comparisons focused on morbidity and mortality after hepatic resection.

### Quality of the included studies

The quality of the nonrandomized studies was assessed using the NOS, and the scores ranged from 7-9, indicating that these studies were of high quality (Tables 1 and 2).

**Table 1:** Interventions of clinical trials included in the meta-analysis

References	Year	Region	Recruitment period	Study design	NOS score	Substratification of treatment(n)	Median FU(m)	3-y OS (%)	5-y OS (%)	3-y DFS (%)	5-y DFS (%)	Primary endpoint
Adam^[11]^	2010	Europe	1995-2009	Cohort	8	NEO(n=169)	28	68	60	46	46	OS+DFS
						SG(n=1302)		73	60	52	42	
Aloysius^[12]^	2007	Europe	2002-2005	Cohort	8	NEO(n=25)	N/A	N/A	N/A	N/A	N/A	M+M
						SG(n=25)						
Araujo^[13]^	2013	America	1998-2007	Cohort	9	NEO(n=175)	58	74	56	32	31	OS+DFS
						SG(n=236)		78	60	44	38	
Ayez^[14]^	2015	Europe	2000-2009	Cohort	9	NEO(n=65)	47	N/A	N/A	N/A	N/A	OS+DFS
						SG(n=154)						
Booney^[15]^	2015	Europe	2000-2011	Cohort	9	NEO(n=693)	31	72	N/A	23	N/A	OS+DFS
						SG(n=608)		74		33		
Boostrom^[16]^	2009	America	2000-2005	Cohort	8	NEO(n=44)	N/A	62	48	20	20	OS+DFS
						SG(n=55)		63	45	32	32	
Cucchetti^[17]^	2012	Europe	2001-2009	Cohort	9	NEO(n=125)	N/A	N/A	N/A	N/A	N/A	M+M
						SG(n=117)						
Hewes^[18]^	2007	Europe	1999-2003	Cohort	7	NEO(n=42)	23	62	N/A	N/A	N/A	OS+DFS
						SG(n=45)		80				
Lubezky^[19]^	2009	Asia	2002-2005	Cohort	8	NEO(n=37)	30.1	70	N/A	50	N/A	OS+DFS
						SG (n=19)	29.2	84		49		
Nordlinger^[20]^	2013	Europe	2000-2004	RCT	9	NEO(n=182)	102	N/A	51.2	38.2	N/A	OS+DFS
						SG (n=182)			47.8	30.3		
Oh^[21]^	2013	Asia	2003-2010	Cohort	7	NEO(n=15)	29	44.0	N/A	37.5	N/A	OS+DFS
						SG (n=15)		55.7		45		
Pinto^[22]^	2012	America	1996-2000	Cohort	8	NEO(n=334)	N/A	59	43	20	13	OS+DFS
						SG (n=342)		71	55	38	26	
Scartozzi^[23]^	2011	Europe	2000-2004	Cohort	9	NEO(n=60)	51	N/A	N/A	N/A	N/A	OS+DFS
						SG (n=44)						
Schreckenbach^[24]^	2015	Europe	2002-2011	Cohort	9	NEO(n=117)	24	N/A	N/A	N/A	N/A	OS+DFS
						SG (n=71)						
Scoggins^[25]^	2009	America	1996-2006	Cohort	7	NEO(n=112)	32	N/A	N/A	N/A	N/A	OS+DFS
						SG (n=74)						
Spelt^[26]^	2012	Europe	2000-2009	Cohort	8	NEO(n=97)	N/A	N/A	N/A	N/A	N/A	M+M
						SG (n=36)						
Tanaka^[27]^	2003	Asia	1985-1999	Cohort	7	NEO(n=48)	23	67.0	83.9	N/A	N/A	OS+DFS
						SG (n=23)		51.8	21.7			
Zhu^[28]^	2014	Asia	2000-2010	Cohort	8	NEO(n=121)	N/A	N/A	N/A	N/A	N/A	OS+DFS
						SG (n=345)						

***OS*** overall survival, ***DFS*** disease-free survival, ***RFS*** recurrence free survival, ***NOS*** Newcastle-Ottawa scale, ***N*** number, ***FU*** follow up, ***M+M*** morbidity and mortality, ***N/A*** not available

***NEO*** neo-adjuvant chemotherapy, ***SG*** surgery alone

**Table 2:** Characteristics of clinical trials included in the meta-analysis

References	Substratification of treatment(n)	Tumor size(mm)	Multiple tumors(n)	Primary site(rectum)	Primary N stage(+)(n)	Synchronous CRLM(n)	Resection type(major)	High risk	Post-chemo (n)	R1 resection (n)	Complication (n)
Adam^[11]^	NEO(n=169)	N/A *	N/A	58	N/A	43	N/A	N/A	N/A	20	62
	SG(n=1302)			466		308				117	312
Aloysius^[12]^	NEO(n=25)	35(25-65)	N/A	N/A	N/A	N/A	N/A	N/A	N/A	N/A	17
	SG(n=25)	40(28-70)									5
Araujo^[13]^	NEO(n=175)	25(17-43)	125*	128	115	153	94	97*	175	22	67
	SG(n=236)	30(20-50)	123	173	134	114	123	74	236	34	92
Ayez^[14]^	NEO(n=65)	26(20-38)		36	46	50*	N/A	N/A	N/A	10	
	SG(n=154)	32(22-44)		70	61	59				21	
Booney^[15]^	NEO(n=693)	N/A	426*	N/A	196	N/A	N/A	N/A	471	N/A	N/A
	SG(n=608)		250		187				413		
Boostrom^[16]^	NEO(n=44)	30	N/A	N/A	28	24	N/A	N/A	N/A	1	N/A
	SG(n=55)	47			29	24				2	
Cucchetti^[17]^	NEO(n=125)	38±24	N/A*	N/A	N/A	94	59	53*	N/A	8	22
	SG(n=117)	40±22				32	40	26		5	15
Hewes^[18]^	NEO(n=42)	N/A	N/A	N/A	N/A	25*	N/A	N/A	N/A	N/A	28
	SG(n=45)					7					9
Lubezky^[19]^	NEO(n=37)	38	N/A*	12	26	N/A	N/A	N/A	N/A	N/A	12
	SG (n=19)	34		5	25						3
Nordlinger^[20]^	NEO(n=182)	N/A	90	84	101	61	N/A	N/A	N/A	N/A	25
	SG (n=182)		86	68	110	67					27
Oh^[21]^	NEO(n=15)	25	N/A	3	12	N/A	N/A	5	0	N/A	1
	SG (n=15)	18		8	13			4	15		1
Pinto^[22]^	NEO(n=334)	33.1±19*	N/A	93	200	234	146	N/A	167	25	58
	SG (n=342)	33.9±20		105	171	125	129		174	18	47
Scartozzi^[23]^	NEO(n=60)	N/A*	20	N/A	N/A	N/A	N/A	5	N/A	N/A	N/A
	SG (n=44)		17					7			
Schreckenbach^[24]^	NEO(n=117)	N/A	N/A*	48	80	87*	71	29	N/A	N/A	N/A
	SG (n=71)			28	44	26	32	17			
Scoggins^[25]^	NEO(n=112)	N/A	N/A	29	92	19	50	N/A	N/A	7	N/A
	SG (n=74)			18	65	9	30			5	
Spelt^[26]^	NEO(n=97)	N/A	N/A	N/A	77	65	59	N/A	N/A	23	61
	SG (n=136)				91	70	80			25	86
Tanaka^[27]^	NEO(n=48)	41(9-160)	N/A	12	N/A	33	39	N/A	N/A	N/A	N/A
	SG (n=23)	53(8-200)		9		18	23				
Zhu^[28]^	NEO(n=121)	60(25-200)	N/A	40	82	61	N/A	N/A	N/A	N/A	25
	SG (n=345)	35(10-180)		34	227	202					47

***Chemo*** chemotherapy, ***CRLM*** colorectal liver metastases, ***NEO*** neo-adjuvant chemotherapy, ***SG*** surgery alone, * significant difference, ***N/A*** not available

### Long-term survival

**Figure 2:**
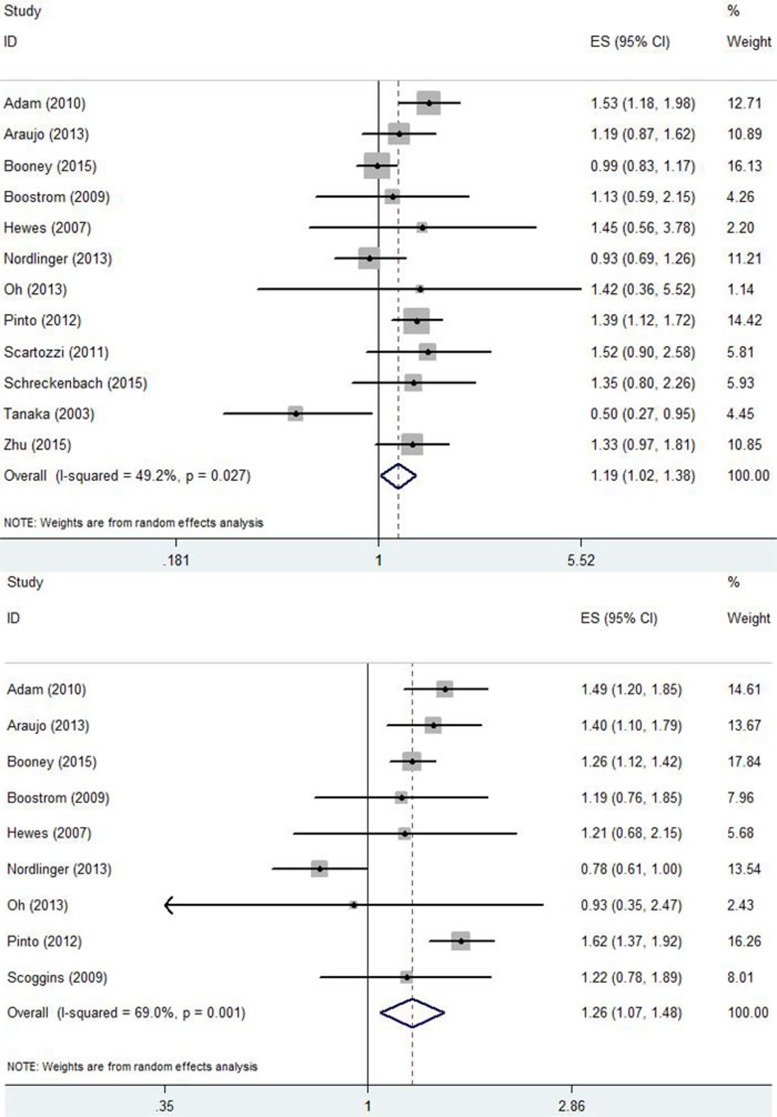
**A.** Forrest plot summarizing the meta-analysis of the 5-year OS rate. **B.** Forrest plot summarizing the meta-analysis of the 5-year DFS rate.

Thirteen studies compared the 3-year OS rate of NEO compared with SG [11, 13, 15, 16, 18–23, 25, 28]. The pooled HR for 3-year OS of NEO calculated using a random-effects model was 1.19 (95% CI: 1.03-1.37; *p* = 0.02; *I*^2^ = 20.7%, *p* = 0.23)(Supplementary Figure 1a). Nine studies compared the 5-year OS rate between NEO and SG [11, 13, 15, 16, 18, 20–23], and two studies found that the 5-year OS rates significantly differed between NEO and SG [22, 27]. The pooled HR for the 5-year OS of NEO calculated using a random-effects model was 1.19 (95% CI: 1.02-1.38; *p* = 0.03; *I*^2^ = 49.2%, *p* = 0.03)(Figure 2a). Ten studies compared the 3-year DFS rate for NEO and SG [11, 13, 15, 16, 18–22, 25]. Two studies found that the 3-year DFS rate was significantly differed between NEO and SG [13, 15]. The pooled HR for the 3-year DFS of NEO calculated using the random-effects model was 1.28 (95% CI: 1.10-1.50; *p* = 0.00; *I*^2^ = 60.6%, *p* = 0.01)(Supplementary Figure 1b). Nine studies compared the 5-year DFS rate for NEO and SG [11, 13, 15, 16, 18, 20–22, 25]. The pooled HR for the 5-year DFS of NEO calculated using the random-effects model was 1.26 (95% CI: 1.07-1.48; *p* = 0.01; *I*^2^ = 69.0%, *p* = 0.00) (Figure 2b).

An additional analysis was also performed to determine whether there was a survival difference among patients depending upon the disease characteristics. The pooled HR for primary lymph node status (positive *vs*. negative) was 1.55 (95 % CI 1.27-1.88, *p* = 0.000; *I*^2^ = 43.8%, *p* = 0.15) (Supplementary Figure 2a), the pooled HR for CEA (>5 *vs*. ≤5 ng/nL) was 1.60 (95 % CI 1.22-2.09, *p* = 0.00; *I*^2^ = 17.9%, *p* = 0.27) (Supplementary Figure 2b), the pooled HR for interval of diagnosis (synchronous *vs*. metachronous) was 1.38 (95 % CI 1.13-1.69, *p* = 0.00; *I*^2^ = 0%, *p* = 0.77) (Supplementary Figure 2c), the pooled HR for tumor size (>5 *vs*. ≤5cm) was 1.39 (95 % CI 1.10-1.76, *p* = 0.01; *I*^2^ = 44.4%, *p* = 0.15) (Supplementary Figure 2d), the pooled HR for surgical margin (positive *vs*. negative) was 1.17 (95 % CI 0.64-2.14, *p* = 0.61; *I*^2^ = 16.7 %, *p* = 0.27) (Supplementary Figure 2e).

### Factors contributing to a high or low risk of recurrence

Four studies identified factors contributing to a high risk of recurrence and compared the 5-year OS rate of NEO and SG [13, 14, 24, 28]. The pooled HR for 5-year OS of NEO calculated using a random-effects model was 0.69 (95% CI: 0.55-0.87; *p* = 0.000; *I*^2^ = 0.0%, *p* = 0.48) (Supplementary Figure 3a). The same four studies identified factors contributing to a low risk of recurrence and compared the 5-year OS rate of NEO and SG [13, 14, 24, 28]. The pooled HR for 5-year OS of NEO calculated using a random-effects model was 1.10 (95% CI: 0.79-1.54; *p* = 0.58; *I*^2^ = 34.8%, *p* = 0.20) (Supplementary Figure 3b).

### Treatment-related complications

Nine studies presented data on complications related to hepatic resection in NEO and SG [12, 13, 17, 19, 21, 22, 25, 26, 28]. The pooled overall OR for NEO was 0.94 (95% CI: 0.89-0.96; *p* = 0.03; *I*^2^ = 62.3%, *p* = 0.01), indicating that neoadjuvant chemotherapy increased the incidence of post-operative complication rate after hepatic resection, as compared to SG (Supplementary Figure 4).

### R1 resection rate

Nine studies presented data on the R1 rate after hepatic resection in NEO and SG [11, 13, 14, 16, 17, 19, 22, 25, 26]. The pooled overall OR for NEO resection was 0.98 (95% CI: 0.95-1.00; *p* = 0.10; *I*^2^ = 0.0%, *p* = 0.76), indicating that neoadjuvant chemotherapy didn not increase R1 rate after hepatic resection, as compared to SG (Supplementary Figure 5).

### Meta-regression

To investigate the effects of regional characteristic on HR estimates, a meta-regression analysis was conducted with subgroups. No statistical significant differences were identified for the treatment effects in the various subgroups. The values of p for the 3-year DFS, 5-year DFS, 3-year OS, 5-year OS and treatment-related complications were 0.47, 0.31, 0.49, 0.68 and 0.74, respectively.

### Sensitivity analysis

Significant heterogeneity was observed for the 3-year, 5-year DFS rate and treatment-related complication rate among the included studies. With respect to the DFS rate, the results reported by Nordlinger et al [20]. differed significantly from the others included studies, which likely contributed to the heterogeneity. After excluding Nordlinger et al., the pooled HR for the 3-year DFS and 5-year DFS of NEO calculated using a random-effects model were 1.40 (95%CI: 1.28-1.53, *p* = 0.000; *I*^2^ = 4.8%, *p* = 0.40) and 1.38 (95%CI: 1.26-1.51, *p* = 0.00; *I*^2^ = 9.6%, *p* = 0.36). For DFS rate, the results reported by Aloysius et al [12]. differed significantly different from others, which likely contributed to the heterogeneity. After excluding Aloysius et al., the pooled overall OR of NEO was 0.96 (95% CI: 0.90-1.01; *p* = 0.13; *I*^2^ = 20.8%, *p* = 0.26), indicating the rate of treatment-related complications did not differ from SG.

### Power analysis and quality of evidence

Power calculations were performed after all of the studies had been collected using the methodology described by Cafri et al. [9]. A power of 82.2% was determined to detect an HR of 1.36 for the 5-year OS of NEO as compared to SG. GRADE Working Group grades for the evidence were high quality for OS and DFS of all resectable CRLM and high quality for 5-year OS of CRLM patients with high risk factors of recurrence.

### Publication bias

For the meta-analysis, inspection of the formal statistical test revealed no evidence of significant publication bias by inspection of the formal statistical tests. For the 5-year OS of NEO *vs*. SG, the results of Egger's test and Begg's funnel plot were 0.98 and 0.53, respectively, and for the 5-year DFS of NEO *vs*. SG, the results were 0.69 and 0.46, respectively.

## DISCUSSION

The present study is the first meta-analysis to assess whether neoadjuvant chemotherapy impacts on the long-term outcomes of patients with initially resectable CRLM. The present study included seventeen cohorts and one RCT, and provided relatively strong evidence of significant benefit neoadjuvant chemotherapy in terms of survival for CRLM patients at high risk of recurrence. Although there was substantial heterogeneity among the studies, the data reported by Nordlinger et al [20]. likely accounted for the majority of it. The difference between their results and the others may reflect the fact that their participants had a smaller liver disease burden than in other studies. For example, in Nordlinger's study more than 50% of patients had only a single metastatic lesion while more than 25% of patients had only two tumors. This could drive heterogeneity of the 3-year and 5-year DFS rate.

Neoadjuvant chemotherapy appeared to negatively impact survival of all patients with resectable CRLM. However, the NEO cohorts had a heavier diseas burden. The patients in NEO had more and larger tumors and more synchronous liver metastases, resulting in a larger number of high-risk patients. It therefore appears that the two groups being compared in these studies were mismatched with respect to many factors. Consequently, most of enrolled studies were in essence comparing less ever with more severe disease, rather than the effect of neo-adjuvant chemotherapy. The only patients found to benefit from neoadjuvant chemotherapy prior to hepatic resection were those with factors indicating a high-risk of recurrence.

Hepatic resection in patients who have already been exposed to systemic chemotherapy is becoming increasingly common in surgical practice [4]. An international panel recommended that the majority of CRLM patients should be treated up front with chemotherapy, irrespective of the initial resectability status of their metastases [29]. One the theoretical advantages of neoadjuvant chemotherapy in the setting of resectable CRLM is that progression while on neoadjuvant chemotherapy would indicate poor disease biology that should be precluded unnecessary resection. On the other hand, a response to chemotherapy may guide the administration of post-operative chemotherapy and the treatment of undetected distant microscopic metastases (aiming to reduce the risk of disease recurrence after resection [19, 23, 30].

The treatment paradigm for CRLM is rapidly shifting to a more personalized approach so as to execute precision medicine [31]. In a large, non-randomized study, patients exhibiting factors associated with a high risk of recurrence gained more benefit from adjuvant therapy than those with factors suggesting a low risk of recurrence [32]. These factors were independent characteristics relating to the features of the liver metastases. Several prognostic scoring models based on those factors may be predictive of recurrence and survival [33–36]. The most widely used and validated clinical risk scores were described by Fong et al. and Nordlinger et al [33, 34]. Based on these scores, four studies identified factors associated with a high risk of recurrence [13, 14, 24, 28]. However, the prognostic significance of the majority of these factors was determined at a time when effective cytotoxic agents were not available. Consequently, although most of these factors are still routinely used, their utility as prognostic indicators in the era of modern chemotherapy is uncertain and should be reassessed. This suggests there is a need to develop new oncological criteria that selects candidates of neoadjuvant chemotherapy. For example, liquid biopsy can predict the liver metastasis disease burden and complement RECIST measurement [37, 38]. KRAS mutation status is a prognostic factor in patients undergoing resection of CRLM, irrespective of chemotherapy regimen [39]. Resection margin is also becoming a focus of attention and reflects a more aggressive surgical strategy [40]. This approach has significant potential to be integrated into the evaluation of patients undergoing neoadjuvant chemotherapy for CRLM.

New chemotherapeutic agents, including irinotecan, oxaliplatin, and the biologic agent bevacizumab, have yielded improved response rates in the treatment of CRLM [41]. Recent data suggested that conflicting results exist regarding the risk of morbidity and mortality associated with preoperative systemic chemotherapy using new agents [25, 42, 43]. Oxaliplatin has been linked to development of hepatic sinusoidal obstruction, while irinotecan is associated with periportal inflammation and steatohepatitis [44, 45]. In addition, when patients in one study received a median of six cycles of neoadjuvant FOLFOX-4 chemotherapy for colorectal liver metastases, it was found that the more cycles of preoperative chemotherapy a patient received, the more chemotherapy-related liver injury was likely to be induced [12]. This may also drive the heterogeneity of hepatic resection related complications. In the present study, neo-adjuvant chemotherapy did not increase morbidity and mortality after hepatic resection. Compared with SG, the pooled overall OR of NEO was 0.96 (95% CI: 0.90-1.01; *p* = 0.13; *I*^2^ = 20.8%, *p* = 0.26). This suggested that preoperative chemotherapy seems to be safe when performing curative hepatic resection for hepatic metastases.

There were several limitations to this meta-analysis that should be taken into consideration. First, it is difficult to draw accurate and consistent conclusions from different protocols of neoadjuvant chemotherapy. Second, most of enrolled studies were retrospective in design and only one study was a randomized controlled trial. Third, CRLM represents a heterogeneous disease in that variations are possible in the number of metastases and the size, location, and most importantly, biological characteristics of the tumors and the proteins they express. There was a significant bias in the two mismatched for most of enrolled studies. It is therefore difficult to judge whether neoadjuvant chemotherapy provides benefit for all resectable CRLM. Finally, the criteria used to assess factors associated with a high risk of recurrence were not standard or convincing as indicators for selecting candidates of neoadjuvant chemotherapy.

In sum, the evidence presented suggests neoadjuvant chemotherapy could improve survival of patients with initially resectable CRLM patients and a high risk factors of recurrence. Further study of neoadjuvant chemotherapy for this subgroup is warranted. Moreover, the efficacy of neoadjuvant chemotherapy should be investigated while taking into account both conventional clinicopathological factors and the molecular factors to define tumor biology.

## SUPPLEMENTARY MATERIAL FIGURES



## References

[1] Siegel R, Ma J, Zou Z, Jemal A (2014). Cancer statistics, 2014. CA Cancer J Clin.

[2] Vigano L, Russolillo N, Ferrero A, Langella S, Sperti E, Capussotti L (2012). Evolution of long-term outcome of liver resection for colorectal metastases: analysis of actual 5-year survival rates over two decades. Ann Surg Oncol.

[3] Adam R, De Gramont A, Figueras J, Guthrie A, Kokudo N, Kunstlinger F, Loyer E, Poston G, Rougier P, Rubbia-Brandt L, Sobrero A, Tabernero J, Teh C, Van Cutsem E (2012). The oncosurgery approach to managing liver metastases from colorectal cancer: a multidisciplinary international consensus. Oncologist.

[4] Chua TC, Saxena A, Liauw W, Kokandi A, Morris DL (2010). Systematic review of randomized and nonrandomized trials of the clinical response and outcomes of neoadjuvant systemic chemotherapy for resectable colorectal liver metastases. Ann SurgOncol.

[5] Wells G, Shea B, O'connell D, Peterson J, Welch V (2000). The Newcastle-Ottawa Scale (NOS) for assessing the quality of nonrandomized studies in metaanalyses. 3rd symposium on systematic reviews: beyond the basics 3-5.

[6] Guyatt GH, Oxman AD, Vist GE, Kunz R, Falck-Ytter Y, Alonso-Coello P, Schunemann HJ (2008). GRADE: an emerging consensus on rating quality of evidence and strength of recommendations. BMJ.

[7] Tierney JF, Stewart LA, Ghersi D, Burdett S, Sydes MR (2007). Practical methods for incorporating summary time-toevent data into meta-analysis. Trials.

[8] Deeks JJ HJ Analysing data and undertaking meta-analyses 2011.

[9] Cafri G, Kromrey JD, Brannick MT (2009). A SAS macro for statistical power calculations in meta-analysis. Behav Res Methods.

[10] Zhou JG, Tian X, Wang X, Tian JH, Wang Y, Wang F, Zhang Y, Ma H (2015). Treatment on advanced NSCLC: platinum-based chemotherapy plus erlotinib or platinum-based chemotherapy alone? A systematic review and meta-analysis of randomised controlled trials. Med Oncol.

[11] Adam R, Bhangui P, Poston G, Mirza D, Nuzzo G, Barroso E, Ijzermans J, Hubert C, Ruers T, Capussotti L, Ouellet JF, Laurent C, Cugat E, Colombo PE, Milicevic M (2010). Is perioperative chemotherapy useful for solitary, metachronous, colorectal liver metastases?. Ann Surg.

[12] Aloysius MM, Zaitoun AM, Beckingham IJ, Neal KR, Aithal GP, Bessell EM, Lobo DN (2007). The pathological response to neoadjuvant chemotherapy with FOLFOX-4 for colorectal liver metastases: a comparative study. Virchows Arch.

[13] Araujo R, Gonen M, Allen P, Blumgart L, DeMatteo R, Fong Y, Kemeny N, Jarnagin W, D'Angelica M (2013). Comparison between perioperative and postoperative chemotherapy after potentially curative hepatic resection for metastatic colorectal cancer. Ann Surg Oncol.

[14] Ayez N, van der Stok EP, Grunhagen DJ, Rothbarth J, van Meerten E, Eggermont AM, Verhoef C (2015). The use of neo-adjuvant chemotherapy in patients with resectable colorectal liver metastases: Clinical risk score as possible discriminator. Eur J Surg Oncol.

[15] Bonney GK, Coldham C, Adam R, Kaiser G, Barroso E, Capussotti L, Laurent C, Verhoef C, Nuzzo G, Elias D, Lapointe R, Hubert C, Lopez-Ben S, Krawczyk M, Mirza DF (2015). Role of neoadjuvant chemotherapy in resectable synchronous colorectal liver metastasis; An international multi-center data analysis using LiverMetSurvey. J Surg Oncol.

[16] Boostrom SY, Nagorney DM, Donohue JH, Harmsen S, Thomsen K, Que F, Kendrick M, Reid-Lombardo KM (2009). Impact of neoadjuvant chemotherapy with FOLFOX/FOLFIRI on disease-free and overall survival of patients with colorectal metastases. J Gastrointest Surg.

[17] Cucchetti A, Ercolani G, Cescon M, Di Gioia P, Peri E, Brandi G, Pellegrini S, Pinna AD (2012). Safety of hepatic resection for colorectal metastases in the era of neo-adjuvant chemotherapy. Langenbecks Arch Surg.

[18] Hewes JC, Dighe S, Morris RW, Hutchins RR, Bhattacharya S, Davidson BR (2007). Preoperative chemotherapy and the outcome of liver resection for colorectal metastases. World J Surg.

[19] Lubezky N, Geva R, Shmueli E, Nakache R, Klausner JM, Figer A, Ben-Haim M (2009). Is there a survival benefit to neoadjuvant versus adjuvant chemotherapy, combined with surgery for resectable colorectal liver metastases?. WorldJ Surg.

[20] Nordlinger B, Sorbye H, Glimelius B, Poston GJ, Schlag PM, Rougier P, Bechstein WO, Primrose JN, Walpole ET, Finch-Jones M, Jaeck D, Mirza D, Parks RW, Mauer M, Tanis E, Van Cutsem E (2013). Perioperative FOLFOX4 chemotherapy and surgery versus surgery alone for resectable liver metastases from colorectal cancer (EORTC 40983): long-term results of a randomised, controlled, phase 3 trial. Lancet Oncol.

[21] Oh SY, Kim DY, Kim YB, Suh KW (2013). Comparison of oncological outcomes between neoadjuvant and adjuvant chemotherapy combined with surgery for resectable synchronous colorectal liver metastases. J Surg Res.

[22] Pinto MH, Barroso E, de Jong MC, Choti MA, Ribeiro V, Nobre AM, Carvalho C, Pawlik TM (2012). Peri-operative chemotherapy for resectable colorectal liver metastasis: does timing of systemic therapy matter?. J Surg Oncol.

[23] Scartozzi M, Siquini W, Galizia E, Stortoni P, Marmorale C, Berardi R, Fianchini A, Cascinu S (2011). The timing of surgery for resectable metachronous liver metastases from colorectal cancer: Better sooner than later? A retrospective analysis. Dig Liver Dis.

[24] Schreckenbach T, Malkomes P, Bechstein WO, Woeste G, Schnitzbauer AA, Ulrich F (2015). The clinical relevance of the Fong and the Nordlinger scores in the era of effective neoadjuvant chemotherapy for colorectal liver metastasis. Surg Today.

[25] Scoggins CR, Campbell ML, Landry CS, Slomiany BA, Woodall CE, McMasters KM, Martin RC (2009). Preoperative chemotherapy does not increase morbidity or mortality of hepatic resection for colorectal cancer metastases. Ann Surg Oncol.

[26] Spelt L, Hermansson L, Tingstedt B, Andersson R (2012). Influence of preoperative chemotherapy on the intraoperative and postoperative course of liver resection for colorectal cancer metastases. World J Surg.

[27] Tanaka K, Adam R, Shimada H, Azoulay D, Levi F, Bismuth H (2003). Role of neoadjuvant chemotherapy in the treatment of multiple colorectal metastases to the liver. Br J Surg.

[28] Zhu D, Zhong Y, Wei Y, Ye L, Lin Q, Ren L, Ye Q, Liu T, Xu J, Qin X (2014). Effect of neoadjuvant chemotherapy in patients with resectable colorectal liver metastases. Plos One.

[29] Nordlinger B, Van Cutsem E, Gruenberger T, Glimelius B, Poston G, Rougier P, Sobrero A, Ychou M (2009). Combination of surgery and chemotherapy and the role of targeted agents in the treatment of patients with colorectal liver metastases: recommendations from an expert panel. Ann Oncol.

[30] Leichman L (2006). Neoadjuvant chemotherapy for disseminated colorectal cancer: changing the paradigm. J Clin Oncol.

[31] Tran NH, Cavalcante LL, Lubner SJ, Mulkerin DL, LoConte NK, Clipson L, Matkowskyj KA, Deming DA (2015). Precision medicine in colorectal cancer: the molecular profile alters treatment strategies. Ther Adv Med Oncol.

[32] Parks R, Gonen M, Kemeny N, Jarnagin W, D'Angelica M, DeMatteo R, Garden OJ, Blumgart LH, Fong Y (2007). Adjuvant chemotherapy improves survival after resection of hepatic colorectal metastases: analysis of data from two continents. J Am Coll Surg.

[33] Nordlinger B, Guiguet M, Vaillant JC, Balladur P, Boudjema K, Bachellier P, Jaeck D (1996). Surgical resection of colorectal carcinoma metastases to the liver. A prognostic scoring system to improve case selection, based on 1568 patients. Association Francaise de Chirurgie. Cancer.

[34] Fong Y, Fortner J, Sun RL, Brennan MF, Blumgart LH (1999). Clinical score for predicting recurrence after hepatic resection for metastatic colorectal cancer: analysis of 1001 consecutive cases. Ann Surg.

[35] Iwatsuki S, Dvorchik I, Madariaga JR, Marsh JW, Dodson F, Bonham AC, Geller DA, Gayowski TJ, Fung JJ, Starzl TE (1999). Hepatic resection for metastatic colorectal adenocarcinoma: a proposal of a prognostic scoring system. J Am Coll Surg.

[36] Nagashima I, Takada T, Matsuda K, Adachi M, Nagawa H, Muto T, Okinaga K (2004). A new scoring system to classify patients with colorectal liver metastases: proposal of criteria to select candidates for hepatic resection. J Hepatobiliary Pancreat Surg.

[37] Tie J, Kinde I, Wang Y, Wong HL, Roebert J, Christie M, Tacey M, Wong R, Singh M, Karapetis CS, Desai J, Tran B, Strausberg RL, Diaz LJ, Papadopoulos N, Kinzler KW (2015). Circulating tumor DNA as an early marker of therapeutic response in patients with metastatic colorectal cancer. Ann Oncol.

[38] Crowley E, Di Nicolantonio F, Loupakis F, Bardelli A (2013). Liquid biopsy: monitoring cancer-genetics in the blood. Nat Rev Clin Oncol.

[39] Brudvik KW, Kopetz SE, Li L, Conrad C, Aloia TA, Vauthey JN (2015). Meta-analysis of KRAS mutations and survival after resection of colorectal liver metastases. Br J Surg.

[40] Pandanaboyana S, White A, Pathak S, Hidalgo EL, Toogood G, Lodge JP, Prasad KR (2015). Impact of margin status and neoadjuvant chemotherapy on survival, recurrence after liver resection for colorectal liver metastasis. Ann Surg Oncol.

[41] Cui CH, Huang SX, Qi J, Zhu HJ, Huang ZH, Yu JL (2015). Neoadjuvant chemotherapy (NCT) plus targeted agents versus NCT alone in colorectal liver metastases patients: A systematic review and meta-analysis. Oncotarget.

[42] Martin RN, Augenstein V, Reuter NP, Scoggins CR, McMasters KM (2009). Simultaneous versus staged resection for synchronous colorectal cancer liver metastases. J Am Coll Surg.

[43] Reddy SK, Morse MA, Hurwitz HI, Bendell JC, Gan TJ, Hill SE, Clary BM (2008). Addition of bevacizumab to irinotecan- and oxaliplatin-based preoperative chemotherapy regimens does not increase morbidity after resection of colorectal liver metastases. J Am Coll Surg.

[44] Aloia T, Sebagh M, Plasse M, Karam V, Levi F, Giacchetti S, Azoulay D, Bismuth H, Castaing D, Adam R (2006). Liver histology and surgical outcomes after preoperative chemotherapy with fluorouracil plus oxaliplatin in colorectal cancer liver metastases. J Clin Oncol.

[45] Vauthey JN, Pawlik TM, Ribero D, Wu TT, Zorzi D, Hoff PM, Xiong HQ, Eng C, Lauwers GY, Mino-Kenudson M, Risio M, Muratore A, Capussotti L, Curley SA, Abdalla EK (2006). Chemotherapy regimen predicts steatohepatitis and an increase in 90-day mortality after surgery for hepatic colorectal metastases. J Clin Oncol.

