# Supplementary material for: The role of neoadjuvant chemotherapy for resectable colorectal liver metastases: a systematic review and meta-analysis
Source: Oncotarget. 2016 Apr 9;7(24):37277–87. doi: 10.18632/oncotarget.8671 (PMC5095075; doi:10.18632/oncotarget.8671)
Supplement: Supplementary file 1 [file oncotarget-07-37277-s001.pdf]

The role of neoadjuvant chemotherapy for resectable colorectal liver metastases: a systematic review and meta-analysis

Supplementary Material

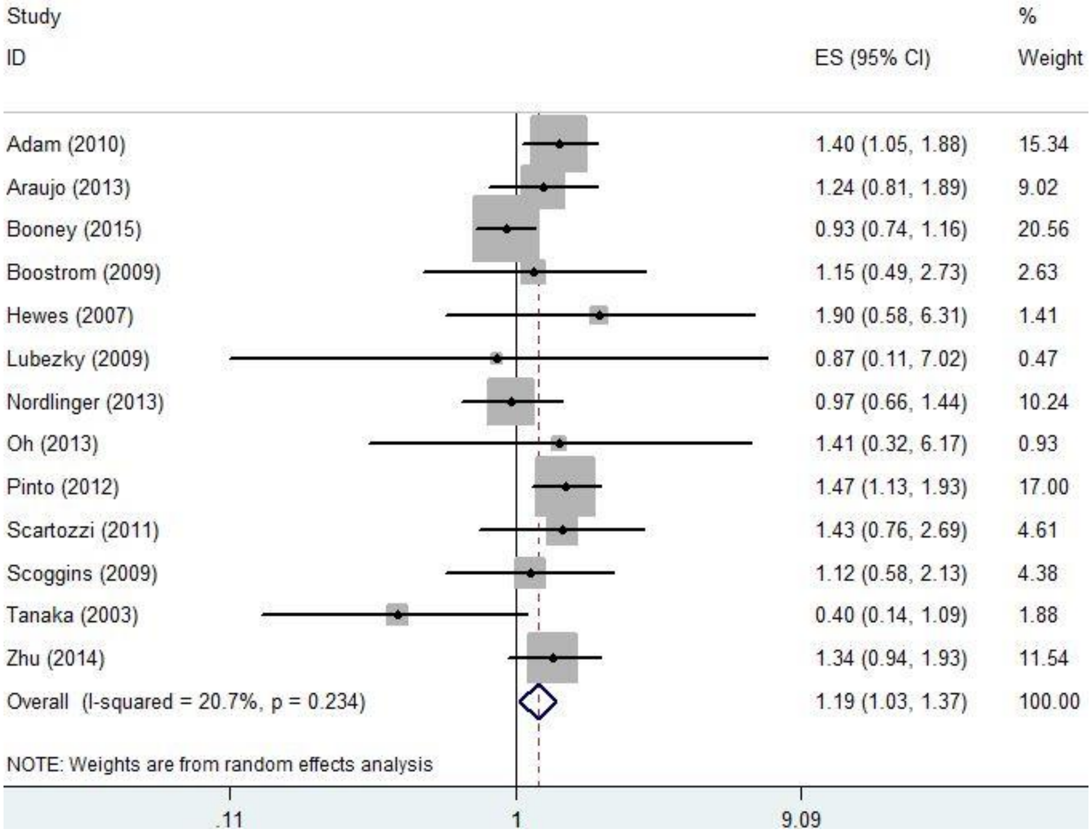

Supplementary Figure1A: Forrest plot summarizing the meta-analysis of the 3-year OS rate

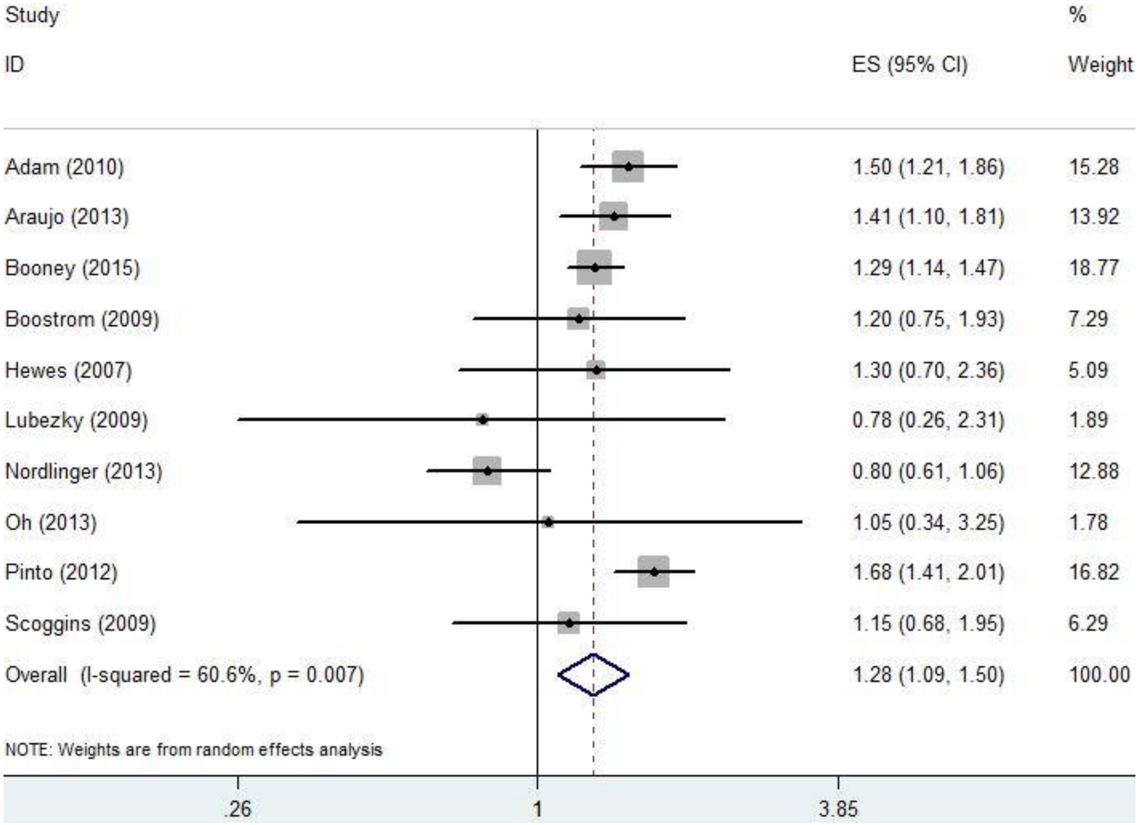

Supplementary Figure1B: Forrest plot summarizing the meta-analysis of the 3-year DFS rate

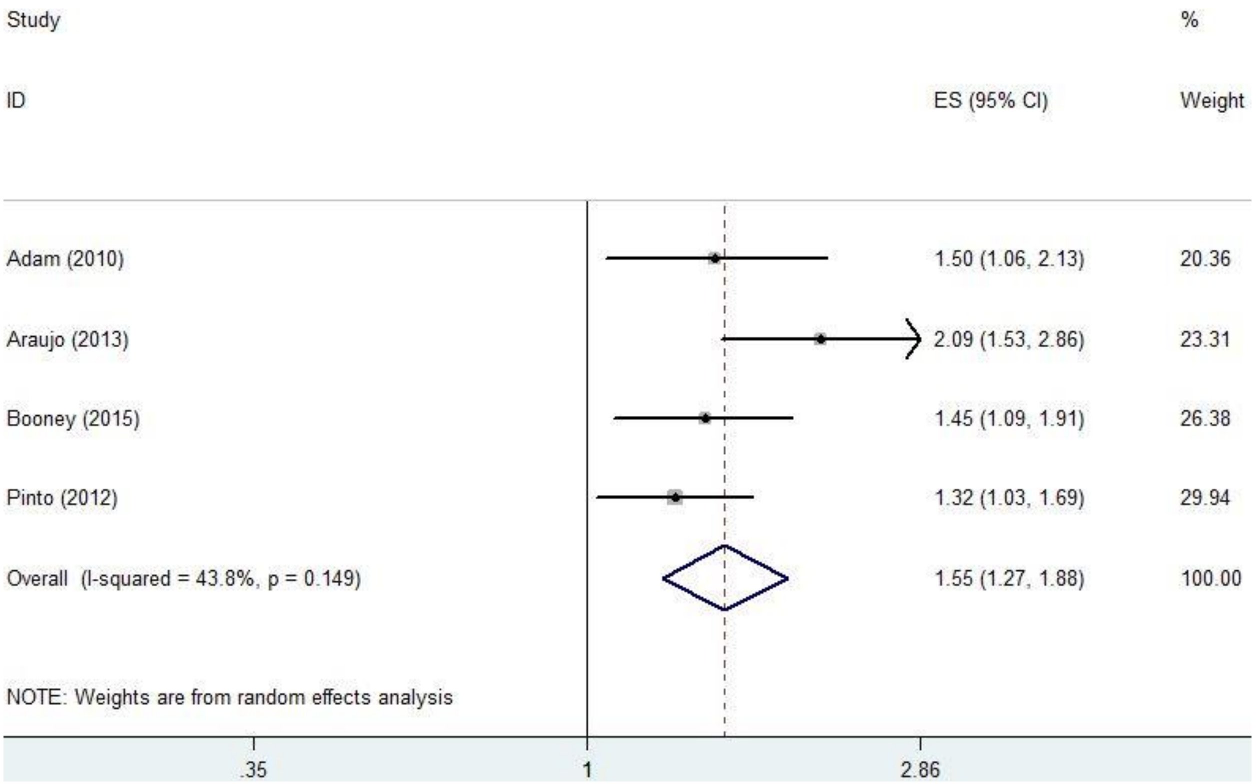

Supplementary Figure 2A: Forrest plot summarizing the meta-analysis of primary lymph node positive

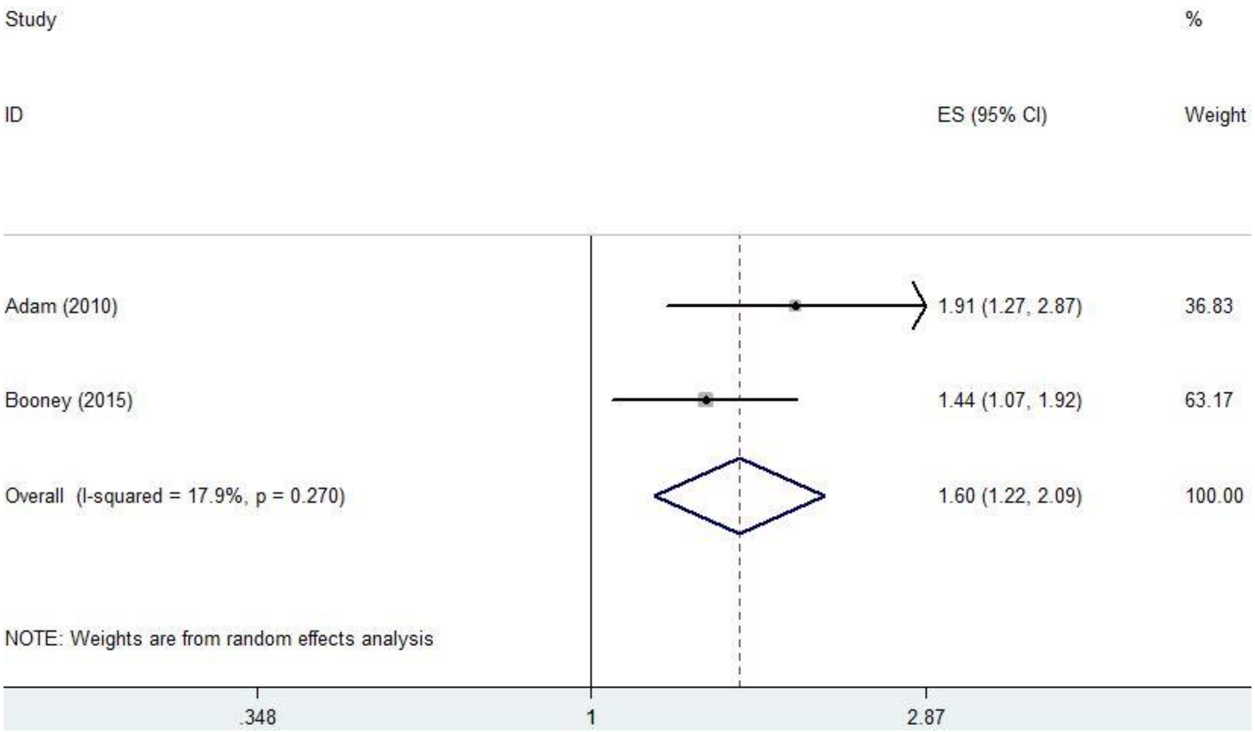

Supplementary Figure 2B: Forrest plot summarizing the meta-analysis of CEA＞5ng/Ml

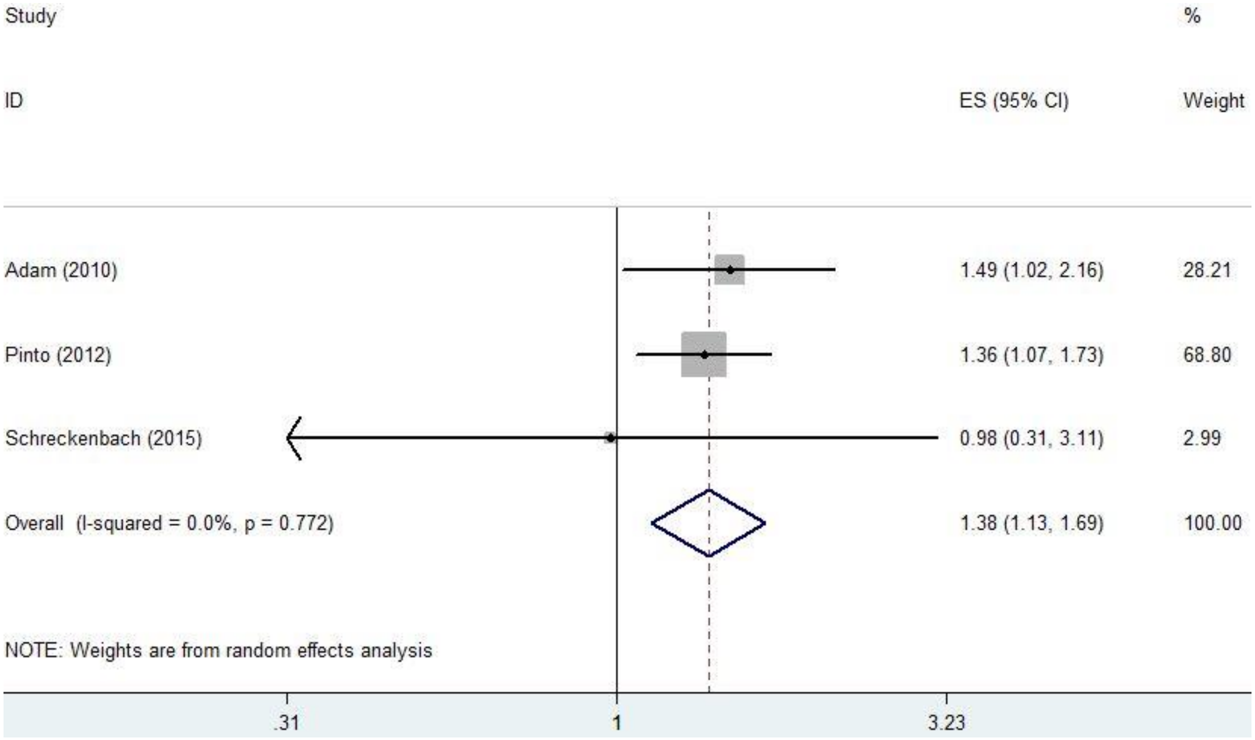

Supplementary Figure 2C: Forrest plot summarizing the meta-analysis of synchronous CRLM

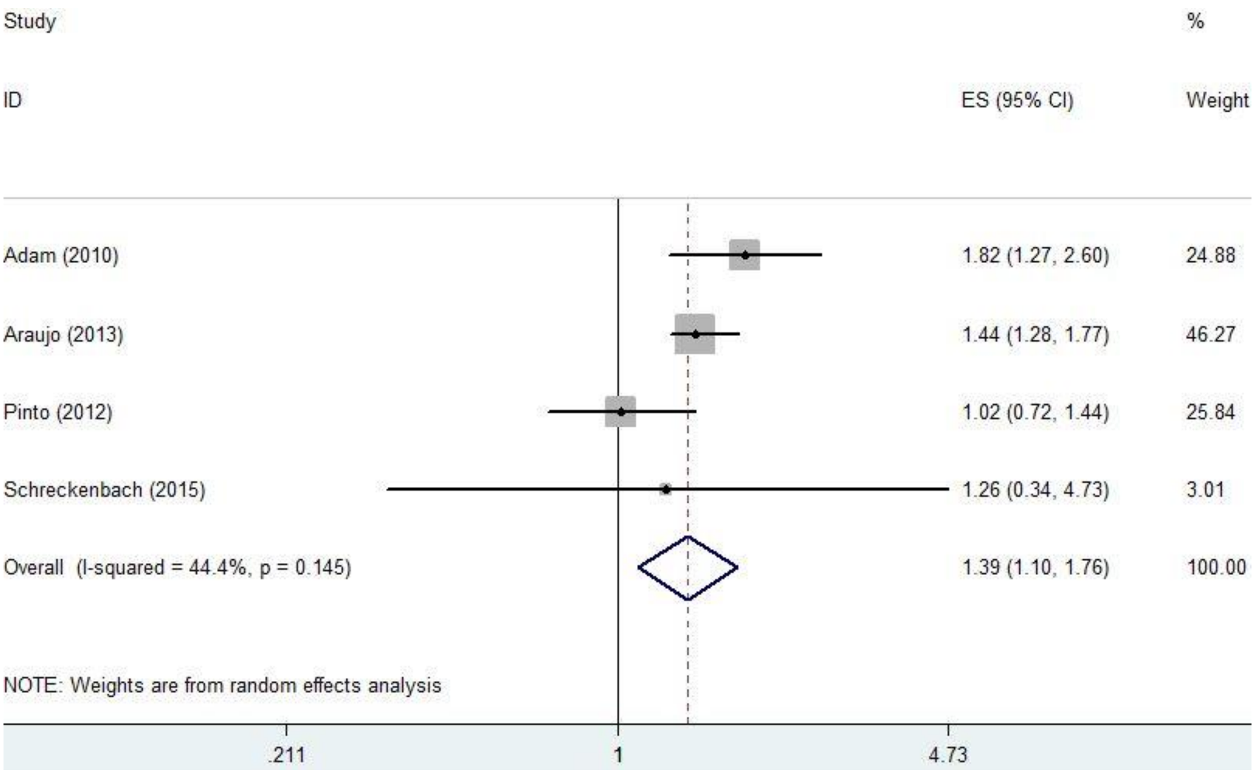

Supplementary Figure 2D: Forrest plot summarizing the meta-analysis of tumor size＞5cm

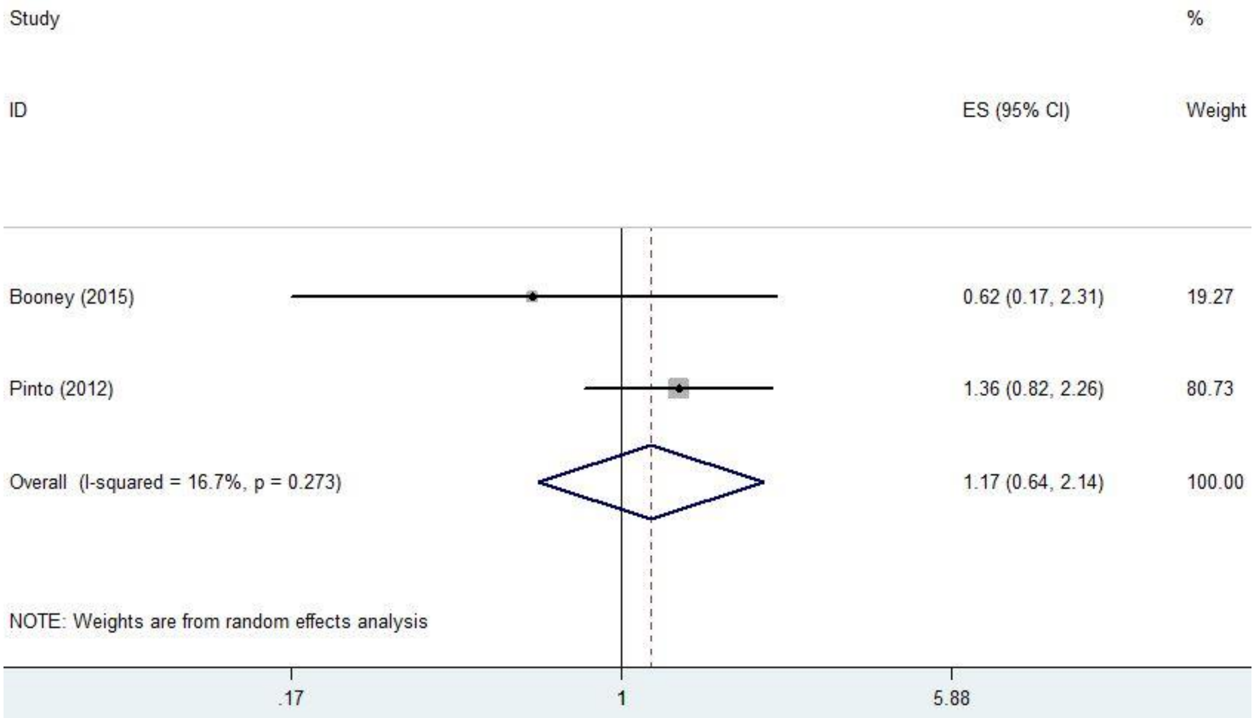

Supplementary Figure 2E: Forrest plot summarizing the meta-analysis of surgical margin positive

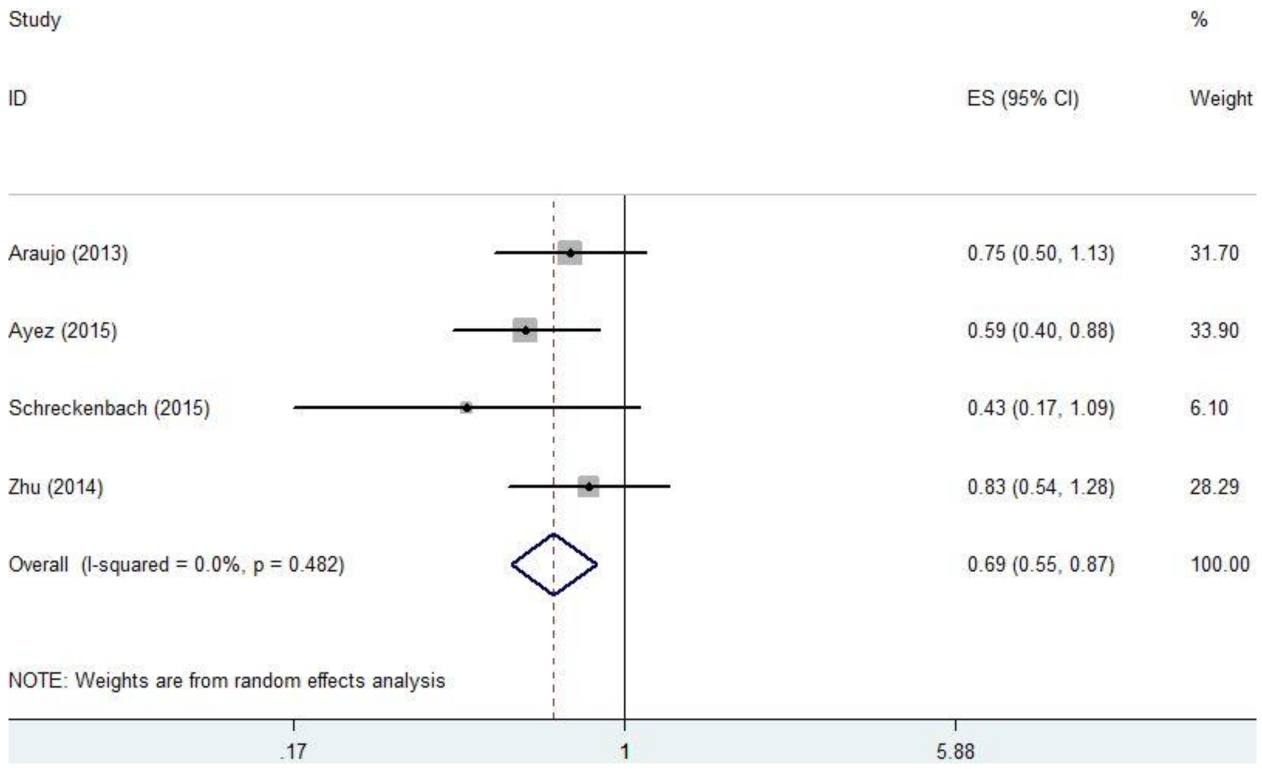

Supplementary Figure 3A: Forrest plot summarizing the OS rate of high risk factors of recurrence

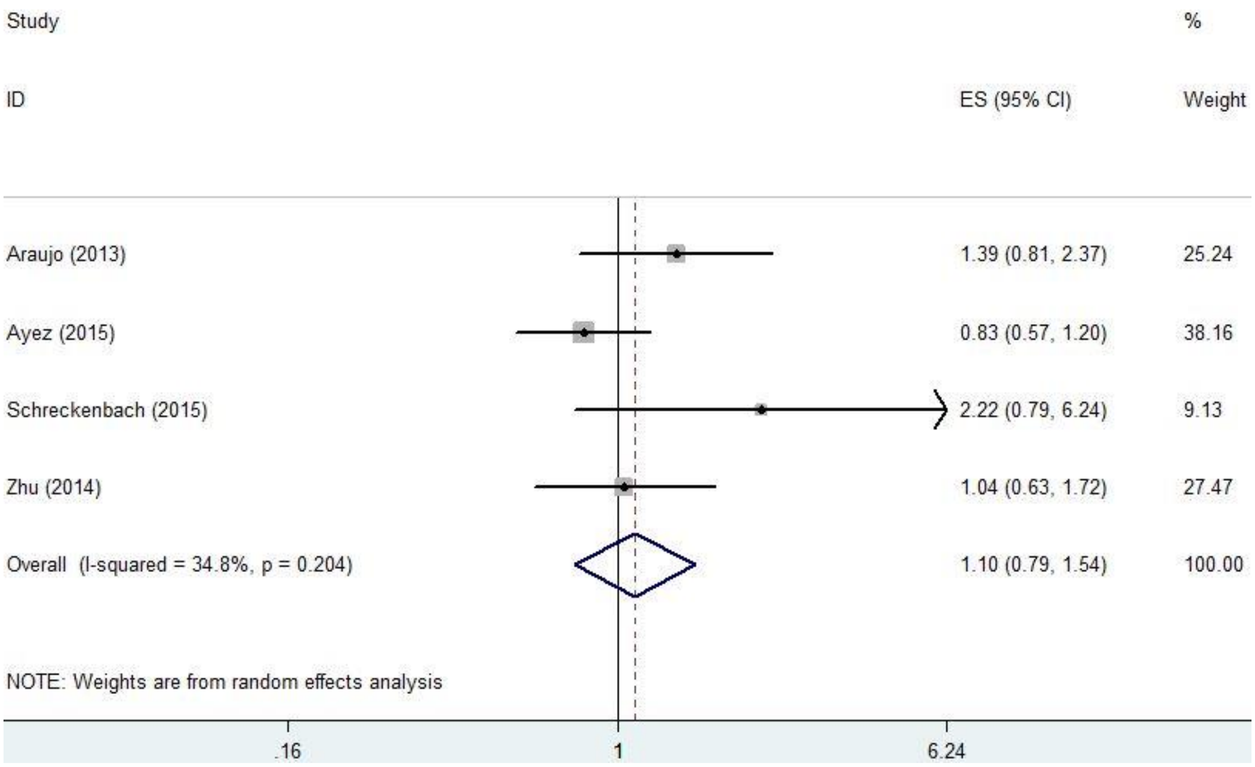

Supplementary Figure 3B: Forrest plot summarizing the OS rate of low risk factors of recurrence

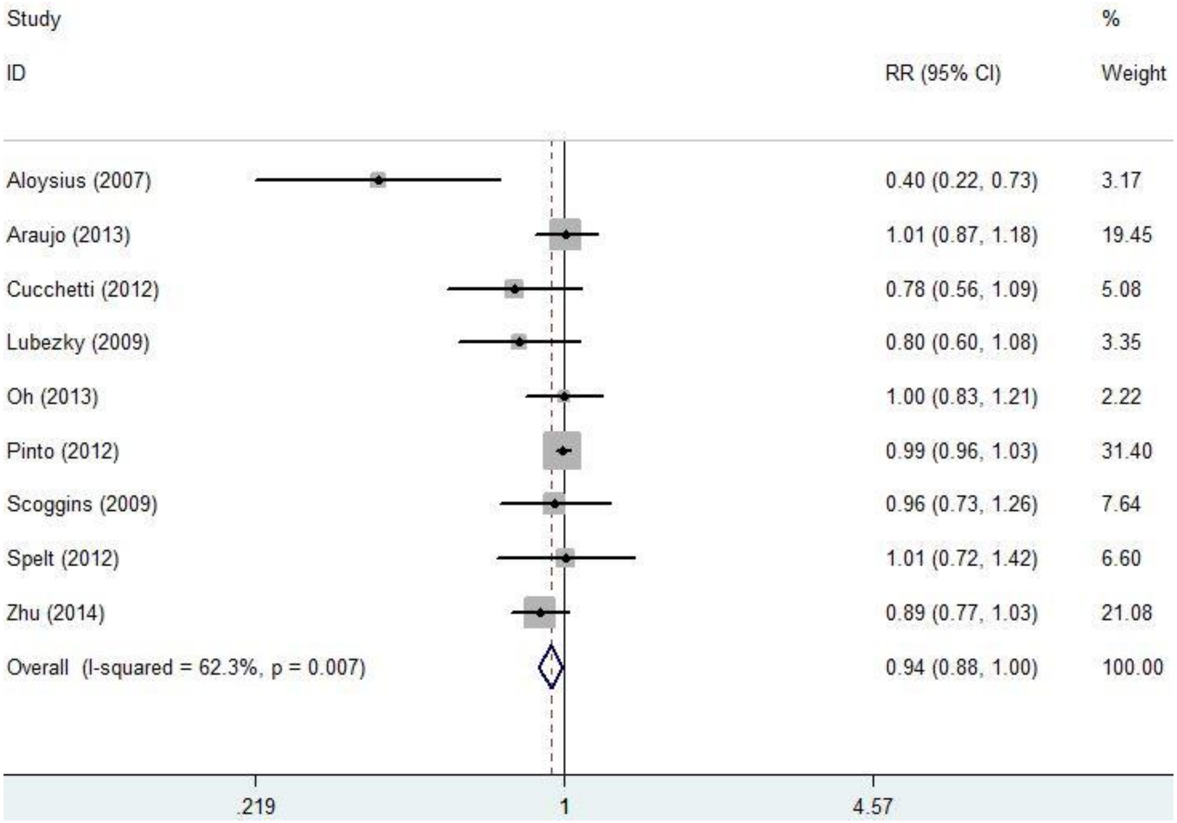

Supplementary Figure 4: Forrest plot summarizing the meta-analysis of treatment-related complication

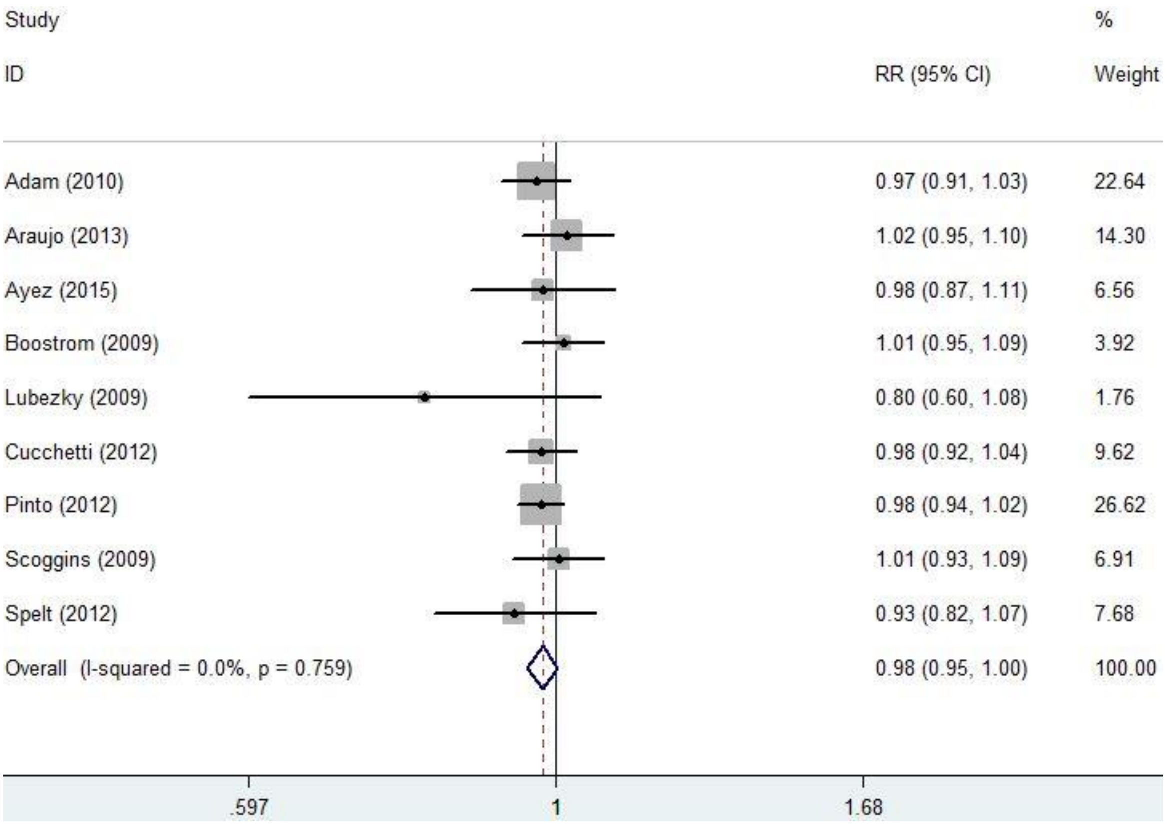

Supplementary Figure 5: Forrest plot summarizing the meta-analysis of R1 resection rate
